# Supplementary material for: Novel predictive factors for patient discomfort and severe cough during bronchoscopy: A prospective questionnaire analysis
Source: PLoS One. 2020 Oct 19;15(10):e0240485. doi: 10.1371/journal.pone.0240485 (PMC7571709; doi:10.1371/journal.pone.0240485)
Supplement: Attachment — Submitted filename: PONE-D-20-07274 editor comments.docx [file pone.0240485.s002.docx]

Editor’s Comments:

Dear Authors,

Thank you for giving me a chance to be an editor of your paper. According to the reviewers’ comments, please revise your manuscript as much as possible. I believe the comments from reviewers would encourage you to refine your paper.
