# Supplementary material for: Novel predictive factors for patient discomfort and severe cough during bronchoscopy: A prospective questionnaire analysis
Source: PLoS One. 2020 Oct 19;15(10):e0240485. doi: 10.1371/journal.pone.0240485 (PMC7571709; doi:10.1371/journal.pone.0240485)
Supplement: Attachment — Submitted filename: to reviewer revise2.docx [file pone.0240485.s004.docx]

Dear Reviewers

Thank you for your kindness to review our manuscript.

We revised the manuscript according to the reviewer's comments point by point.

We are very grateful to your great effort.

Thank you again.

Reviewer #1: The authors have addressed the reviewers' comments in this revision. I only have one more comment. Please update the p-values in Tables 3-7 with more accurate p-values rather than a range, e.g., <0.05.

R: Thank you for your comments. We revised them.

Reviewer #2: The manuscript has been improved by the authors.
However, the problem has been remaining that the difference between cough score 1 (slight cough) and 2 (moderate cough, no need to interrupt procedure) was ill defined. How did the bronchoscopists decide which patients had slight cough or moderate cough?

R: Thank you for your thoughtful comments. We revised them (Fig.2 and Table2) more precisely as follows.

| **Score** | **Cough severity** |
| --- | --- |
| 0 | No cough |
| 1 | A slight cough：go ahead the procedure |
| 2 | Moderate cough: transient interruption of the procedure in the trachea |
| 3 | Severe cough: removal of the bronchoscope from the trachea |

Reviewer #3: (No Response)
