# Supplementary material for: Novel predictive factors for patient discomfort and severe cough during bronchoscopy: A prospective questionnaire analysis
Source: PLoS One. 2020 Oct 19;15(10):e0240485. doi: 10.1371/journal.pone.0240485 (PMC7571709; doi:10.1371/journal.pone.0240485)
Supplement: Attachment — Submitted filename: Response to Reviewer.docx [file pone.0240485.s005.docx]

Dear Reviewer1

Thank you for your kindness to review our manuscript.

We revised the Table3 as follows.

Thank you for your comments.

We are very grateful to your great effort.

Thank you again.

**Table 3. Patient characteristics and univariate analysis for discomfort of bronchoscopy**

| **Factor** | **Total n=172** | **Discomfort n=84** | **Comfort n=88** | ***p value*** |
| --- | --- | --- | --- | --- |
| Age (year) ^a^ | 67.83 ± 13.44 | 64.06 ± 14.10 | 71.43 ±11.77 | < 0.001 |
| Gender (Male/Female), n (%)^d^ | 117/55 (68.0/32.0) | 51/33 (60.7/39.3) | 66/22 (75.0/25.0) | 0.065 |
| Suspected disease |  |  |  |  |
| Lung tumor, n (%)^d^ | 106 (61.6) | 48 (57.1) | 58 (65.9) | 0.305 |
| Interstitial pneumonia, n (%)^d^ | 30 (17.4) | 16 (19.0) | 14 (15.9) | 0.733 |
| Sarcoidosis, n (%)^c^ | 13 (7.6) | 10 (11.9) | 3 (3.4) | 0.044 |
| Infectious disease, n (%)^c^ | 11 (6.4) | 4 (4.8) | 7 (8) | 0.536 |
| Other, n (%)^d^ | 12 (7.0) | 6 (7.1) | 6 (6.8) | 1.000 |
| Weight (kg) ^a^ | 57.60 ± 11.52 | 56.71 ± 11.51 | 58.53 ± 11.53 | 0.628 |
| BMI (kg /m^2^) ^a^ | 22.1 ± 3.35 | 22.43 ± 3.67 | 21.83 ± 3.00 | 0.565 |
| Outpatient, n (%)^c^ | 8 (4.65) | 4 (4.8) | 4 (4.5) | 1.000 |
| Procedure |  |  |  |  |
| BAL, n (%)^d^ | 35 (20.3) | 23 (27.4) | 12 (13.6) | 0.041 |
| TBLB, n (%)^d^ | 28 (16.3) | 18 (21.4) | 10 (11.4) | 0.114 |
| EBUS-GS-TBB, n (%)^d^ | 75 (43.6) | 35 (41.7) | 40 (45.5) | 0.730 |
| EBUS-TBNA, n (%)^d^ | 55 (32.0) | 33 (39.3) | 22 (25.0) | 0.065 |
| EBB, n (%)^d^ | 12 (7.0) | 6 (7.1) | 6 (6.8) | 1.000 |
| TBB, n (%)^c^ | 3 (1.7) | 0 (0) | 3 (3.4) | 0.246 |
| Brushing, n (%)^c^ | 9 (5.2) | 4 (4.8) | 5 (5.7) | 1.000 |
| Observation, n (%)^c^ | 10 (5.8) | 2 (2.4) | 8 (9.1) | 0.100 |
| Treatment, n (%)^c^ | 4 (2.3) | 3 (3.6) | 1 (1.1) | 0.359 |
| Less-experienced bronchoscopist, n (%) ^d^ | 116 (67.4) | 64 (76.2) | 52 (59.1) | 0.024 |
| Drug |  |  |  |  |
| Midazolam and pethidine, n (%)^d^ | 159 (92.4) | 77 (91.7) | 82 (93.2) | 0.930 |
| Midazolam only, n (%)^c^ | 2 (1.2) | 1 (1.2) | 1 (1.1) | 1.000 |
| Pethidine only, n (%)^c^ | 11 (6.4) | 6 (7.1) | 5 (5.7) | 0.763 |
| Total midazolam (mg) ^a^ | 2.70 ± 1.58 | 2.95 ± 1.75 | 2.45 ± 2.36 | 0.050 |
| Total pethidine (mg) ^a^ | 34.59 ± 5.97 | 34.58 ± 6.06 | 34.60 ± 5.92 | 0.977 |
| Examination time (min) ^b^ | 38.48 ± 17.20 | 41.96 ± 17.46 | 35.15 ± 16.36 | 0.009 |
| Cough score ^a^ | 1.07 ± 3.35 | 1.43 ± 1.04 | 0.73 ±0.93 | < 0.001 |
| Δ systolic BP (mmHg) ^a^ | 20.06 ± 27.13 | 25.64 ± 26.60 | 14.74 ± 26.70 | 0.004 |
| Δ Heart rate (/min) ^a^ | 15.02 ± 19.62 | 18.95 ± 19.23 | 11.26 ± 19.35 | 0.006 |
| Questionnaire before bronchoscopy |  |  |  |  |
| Retry (1^st^ time/≧ 2^nd^ time), n (%)^d^ | 135/37 (78.5/21.5) | 65/19 (77.4/22.6) | 70/18 (79.5/20.5) | 0.873 |
| Anxiety ^a^ | 3.54 ± 1.30 | 3.81 ± 1.15 | 3.28 ± 1.39 | 0.016 |

BAL, bronchoalveolar lavage; BMI, body mass index; BP, blood pressure; EBB, endobronchial biopsy; EBUS-GS-TBB, endobronchial ultrasonography with a guide sheath transbronchial biopsy, EBUS-TBNA: Endobronchial ultrasound-guided transbronchial needle aspiration, TBB: transbronchial biopsy, TBLB: transbronchial lung biopsy,

Data are presented as mean ± standard deviation. a: Mann–Whitney U test, b: t-test. c: Fisher’s exact test D:χ^2^ test
